# MicroRNA-378 Promotes Osteogenesis-Angiogenesis Coupling in BMMSCs for Potential Bone Regeneration

**DOI:** 10.1155/2018/8402390

**Published:** 2018-03-01

**Authors:** Bo Zhang, Yali Li, Yang Yu, Jinlong Zhao, Yangzhen Ou, Yu Chao, Binhui Yang, Xiaorui Yu

**Affiliations:** ^1^Department of Biochemistry and Molecular Biology, School of Basic Medical Sciences, Xi'an Jiaotong University Health Science Center, Xi'an Jiaotong University, Xi'an, Shaanxi 710061, China; ^2^3201 Hospital Affiliated to Xi'an Jiaotong University, Hanzhong, Shaanxi 723000, China; ^3^Key Laboratory of Environment and Genes Related to Diseases (Xi'an Jiaotong University), Ministry of Education, Xi'an, Shaanxi 710061, China

## Abstract

Bone tissue regeneration was closely associated with osteogenesis and angiogenesis. The harmonious regulation of osteogenetic and angiogenic growth factors would enhance bone regeneration, while the imbalance of that would lead to local excessive bone formation or vascular mass due to exogenous delivery. Therefore, microRNA is believed to regulate multiple metabolism progress through endogenous signaling pathways on the gene level. In this work, we identified microRNA 378 as a positive regulator of osteogenesis and angiogenesis simultaneously and also observed an increase of microRNA 378 than control in human bone marrow mesenchymal stem cells (hBMMSCs) after osteoblast induction. Besides, osteogenetic and angiogenic gene expression increased simultaneously after overexpression of microRNA 378. Moreover, alizarin red staining and alkaline phosphatase (ALP) staining enhanced, and secretion of vascular endothelial growth factor (VEGF) increased. In this way, we believed miR378 was an ideal target to osteogenesis-angiogenesis coupling for bone regeneration, which provides a potential tool for the gene therapy of bone regeneration.

## 1. Introduction

After transplantation, insufficient blood supply will cause inflammation and necrosis in the huge bone graft, which lead to high failure rate in clinical use [1]. Therefore, angiogenesis is important to bone formation progress in which blood supply induces migration of osteoblasts and mineralization of bone tissue [2, 3]. The imbalance of osteogenesis and angiogenesis will cause local bone tissue overmineralization or blood vessel mass [4].

Multiple endogenous signaling pathways regulate osteogenesis and angiogenesis. Wnt signaling pathway positively regulates osteogenesis in BMMSC and ALP and runt-related transcription factor 2 (Runx2), which are the representative factors in the downstream of osteogenesis-angiogenesis [5–7]. Mitogen-activated protein kinase (MAPK) signaling pathway positively regulates angiogenesis by targeting VEGF expression [8–10]. However, exogenous osteogenetic and angiogenic stimulants would cause imbalance of bone formation, leading to excessive bone formation or vascular leakage at the risk of tumorigenesis [11, 12]. Therefore, it is necessary to increase osteogenesis and angiogenesis with potential, endogenous, and balanced stimulation.

MicroRNAs (miRNAs) are a large class of small, noncoding RNAs that functioned as repressors of gene expression at the level of posttranscriptional regulation. A single miRNA is often involved in several gene regulatory networks which functioned as a checkpoint of different signaling pathways [13–15]. Therefore, we tried to identify a microRNA as osteogenesis-angiogenesis coupling for bone regeneration. From previous reports, miR378, miR214, and miR155 were greatly affected on both osteogenesis and angiogenesis processes. Through target gene prediction, miR378 was chosen as the key factor to regulate both osteogenesis and angiogenesis.

## 2. Methods

### 2.1. Culture of Human BMMSCs

Primary human BMMSCs were isolated as previously described [16]. The sample collection was conformed to protocol approved by the ethical authorities at the 3201 Hospital Affiliated to Xi'an Jiaotong University. The experiment was conducted with the donors' or subjects' understanding and consent, as well as a statement that the responsible Ethical Committee has approved the experiments.

First, healthy bone marrow samples were collected from 5 donors, aged from 18 to 30 years, undergoing the alveolar bone cleft repair by autoilium transplantation. 1 mL bone marrow aspirates were seeded into a 10 cm culture dish and added 20 mL *α*-MEM (Gibco BRL, Gaithersburg, MD) supplemented with 15% FBS (Gibco, BRL), then incubated in 5% CO_2_, 37°C. Cells from 3–5 passages were used in the experiments.

Second, fluorescence-activated cell sorting (FACS) analysis was used for analyzing CD45, CD34, CD14, CD29, CD44, CD90, and CD106 (R&D Systems, Inc.). Multiple colony-derived hMSCs at 2–4 passages were used in our experiments.

### 2.2. Culture of MC3T3

Take 4 mL of *α*-MEM complete medium to the centrifuge tube and preincubate at 37°C for 10 min. The cryopreserved MC3T3 cells (ATCC, USA) removed from the liquid nitrogen were quickly shaken in a 37°C water bath and reshaken quickly. The cells were transferred to the preheated medium and the supernatant was centrifuged. The appropriate amount of *α*-MEM was completely cultured at 37°C 5% CO_2_ incubator. On the next day, the fresh medium was replaced and incubated at 37°C in a 5% CO_2_ incubator. Cells were used in experiments 3 to 5 generations.

### 2.3. Culture of EPCs

Take 8 mL of EGM-2MV (Lonza, Switzerland) medium and warm it in a 37°C water bath. Take rEPC (ATCC, USA) from the liquid nitrogen cans and quickly shake them into the 37°C water bath and shake the cells quickly. Centrifuge discarded supernatant and add the appropriate amount of fresh medium, 37°C, and 5% CO_2_ incubator to continue culture after every 3 days to replace the medium. Cells were used in experiments 3 to 5 generations.

### 2.4. Transfection

hBMMSCs were transfected with miR-378 mimics, miR-378 inhibitor, or negative controls (Guangdong Ruibo, China) using siPORT NeoFX transfection agent (Ambion, Applied Biosystems, USA) and following the manufacturer's instructions. Briefly, the transfection reagent was diluted in *α*-MEM (Gibco BRL) and mixed with 50 nM miR-378 mimic, 100 nM miR-378 inhibitor, or with the same concentration of the miR-negative control. After incubating for 10 min at room temperature, the mixture was dispensed into 6-well plates (Nunc). The BMMSC suspension was added to each flask at a density of 1 × 10^6^ cells/mL and cultured in normal conditions. The media was replaced after a 6-hour transfection. Cells were harvested at different time points (days 2, 7, and 14) after transfection, and levels of RNAs were analyzed by quantitative RT-PCR (qRT-PCR). All reactions were repeated 3 times.

### 2.5. Osteogenic Differentiation

To induce osteoblast differentiation, after reaching 80% confluence, BMMSC and MC3T3 cells were cultured in *α*-MEM supplemented with 5% FBS, 100 nM dexamethasone, 50 *μ*g/mL of ascorbic acid, and 5 mM *β*-glycerophosphate for 14 days. The media were changed every three days. Calcium accumulation was detected by 2% alizarin red staining and dissolved by 1 mL of sodium dodecyl sulfate solution. The light absorption of sodium dodecyl sulfate solution with alizarin red was read at 570 nm with a microplate reader (Bio-TEK Instruments, Winooski, VT, USA). After 7-day and 14-day osteogenetic induction, the cells were washed twice in phosphate-buffered saline (PBS) after fixation in 4% paraformaldehyde for 20 min. ALP staining was determined with the BCIP/NBT Alkaline Phosphatase Color Development Kit (Beyotime Co., Shanghai, China). The expression of osteoblastic genes Runx2, ALP, osteocalcin (OCN), collagen I (Col I), and bone sialoprotein (BSP) was determined by PCR assay.

### 2.6. Matrigel™ Angiography

BMMSC and EPC suspensions were cultured for the third generation for subsequent Matrigel experiments. BMMSCs were cultured for the third generation and then transfected with BMRCs by miR-378 mimetic, miR-378 inhibitor, or negative controls for subsequent experiments.

Matrigel [growth factor-reduced (GFR) Matrigel Matrix, BD, USA] was placed in a freezer and thawed overnight in a 4°C refrigerator overnight. In the experiment, the Matrigel was always placed in the icebox. Add 10 *μ*L of Matrigel to each well of the ibidiangiogenesis slide and cover the lid and allow the slide to stand for about 30 minutes before waiting for gelation. After gelatinization, add 50 *μ*L of cell suspension at a concentration of 1 × 10^6^ cells/mL to each column of the slide, cover, and hold, and after a period of time, all cells will sink to the Matrigel's surface. The number of branches, branch length, node, number of mesh, and mesh area was analyzed.

### 2.7. Quantitative RT-PCR

BMMSCs were cultured in osteogenic medium and harvested in 14-day induction. Osteogenetic marker genes (Runx2, Alp, Bmp2, and osteocalcin) were examined by qRT-PCR in BMMSCs. After BMMSCs were transfected with miR-378 mimics, miR-378 inhibitor, or negative controls, miR-378 level, Runx2, Bmp2, Ocn, VEGF, angiopoietin 1 (Ang1), and Col I were examined by qRT-PCR after BMMSCs were transfected with miR-378 mimics, miR-378 inhibitor, or negative controls. Total cellular RNA was isolated with Trizol reagent (Invitrogen, Carlsbad, CA). Then, isolated RNA was used as a template for cDNA synthesis, and the prepared Superscript II first-strand cDNA synthesis kit was used (Invitrogen Life Technologies, Carlsbad, CA). Real-time PCR was performed according to the manufacturer's protocol. The primers were listed in Table 1. All qRT-PCR tests were repeated three times for each sample.

### 2.8. Enzyme-Linked Immunosorbent Assay (ELISA)

After the BMMSCs were transfected with miR-378 mimics, miR-378 inhibitor, or negative controls, VEGF secretion was analyzed. BMMSCs were cultured with *α*-MEM (Gibco) in 6 h, and then, the medium was replaced with freshly growing medium supplemented with 5.0% serum substitute nu-Serum# (NuS, BD). Samples were collected at 6 h, 12 h, 24 h, 36 h, 48 h, and 60 h. VEGF protein levels in the medium were determined using an ELISA according to the manufacturer's instructions (R&D Corp.). Absorbance was measured at 450 nm with a microplate reader (MTP-800Lab, Corona Electric, Japan). A standard curve was plotted to determine the VEGF concentration. All tests were repeated three times for each sample. The values are expressed as ng/mL.

### 2.9. Western Blot

After BMMSCs were transfected with miR-378 mimics, miR-378 inhibitor, or negative controls, VEGF expression was analyzed. The total protein was extracted from the three groups of cells; the expression level of VEGF was detected by WB.

### 2.10. Statistical Analyses

All experiments have been performed at least in triplicate. Results are expressed in means and standard deviations. Statistical analyses (Student's *t*-tests) have been performed as two sample *t*-tests.

## 3. Results

### 3.1. miR378 Was Involved in Osteogenesis of BMMSCs

**Figure 1:**
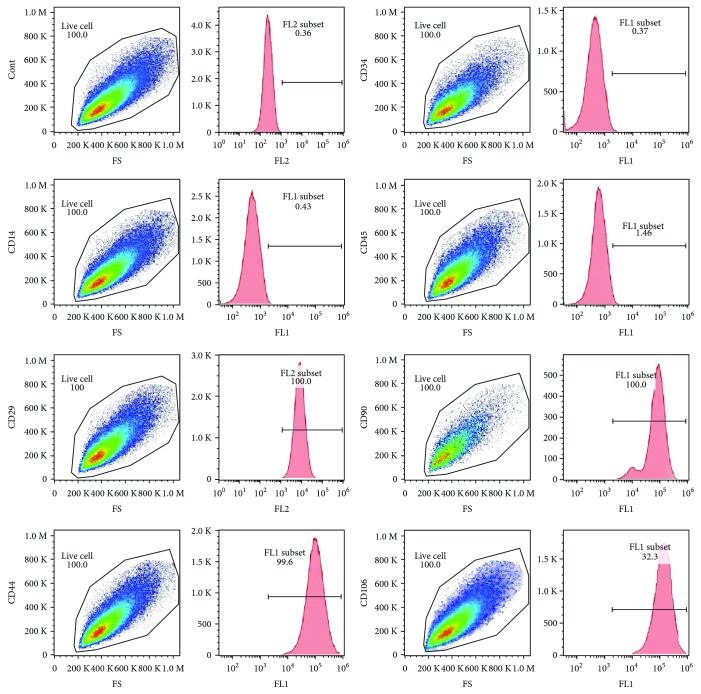
Identification of stem cell-related cell surface markers on hBMMSCs. Detection of cell surface markers was characteristic of mesenchymal stem cells on the three cells. Analyses were performed via flow cytometry, detecting PE- or FITC-conjugated monoclonal antibodies for human CD29, CD44, CD90, CD106, CD14, CD34, CD45, or isotype-matched control IgGs. Cont: control.

**Figure 2:**
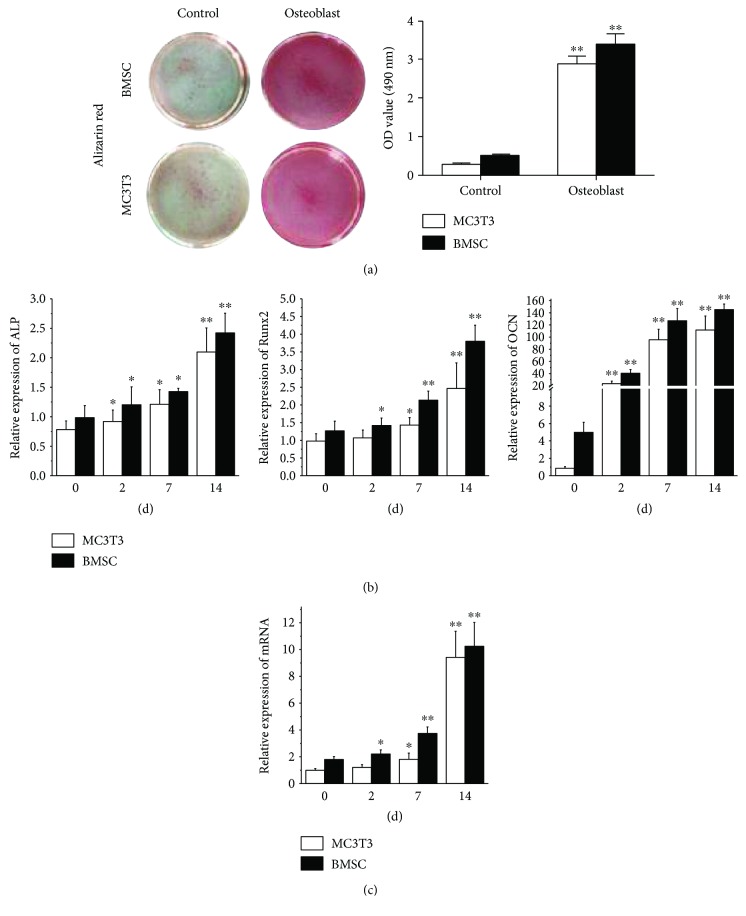
MiR-378 expression with osteoblast differentiation in hBMMSCs and MC3T3 cells. (a) Alizarin red staining was performed at day 14. (b) Osteoblast relative gene was detected by qRT-PCR after 2-day, 7-day, and 14-day osteoblast induction. (c) miR-378 expression was significantly induced during differentiation in hBMMSCs and MC3T3 cells. ^∗^*P* < 0.05 and ^∗∗^*P* < 0.01; *n* = 3.

BMMSCs were cultured to P3 and cell markers were analyzed by FACS analysis. The results showed that BMMSCs positively expressed CD29, CD44, CD90, and CD106 and negatively expressed CD14, CD34, and CD45 (Figure 1). Through target gene prediction analysis, miR378 was predicted as an intersection of Wnt and MAPK signaling pathway. Therefore, we chose miR378 as a regulator to promote osteogenesis and angiogenesis simultaneously. After 14-day osteogenetic induction, alizarin red staining was performed. Compared with MC3T3, BMMSCs with osteogenetic induction showed 3 times stronger staining (Figure 2(a)). And after 7-day and 14-day osteogenetic induction, osteogenetic gene was analyzed by qRTPCR. The results showed that ALP and Runx2 expression of BMMSCs increased to 3- to 4-fold after 7-day induction and more 2- to 3-fold after a 14-day induction. Moreover, OCN expression was 90-fold stronger after the 7-day induction and 140-fold stronger after the 14-day induction (Figure 2(b)). Meanwhile, we also tested the miR378 level. And the result showed that after 7-day and 14-day induction, miR378 expression was significantly higher than control (Figure 2(c)).

### 3.2. Increased Osteogenesis Related to miR378 Positively

**Figure 3:**
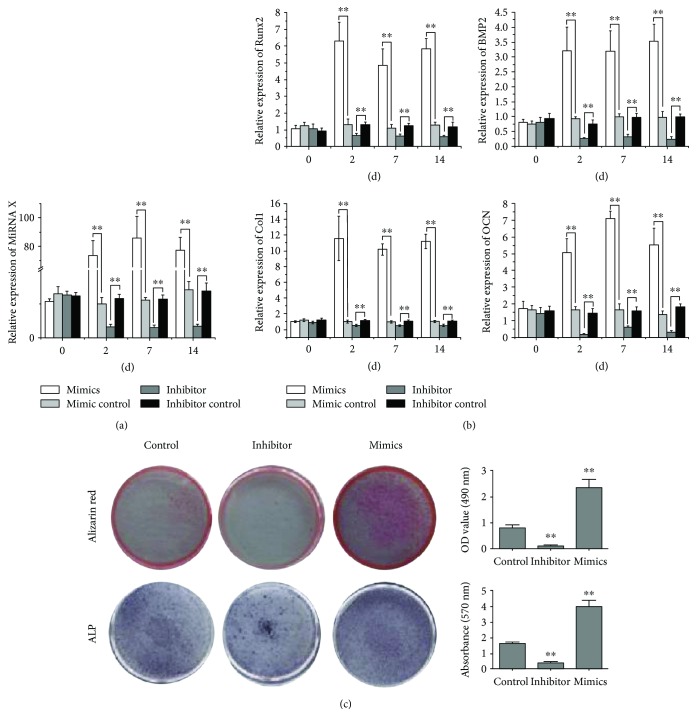
MiR-378 enhanced osteogenesis in vitro. BMMSCs that were transfected with miR-378 mimics, miR-378 inhibitor, or the miR-negative control. (a) MiR-378 expression was enhanced and reduced by transfection of miR-378 mimics and miR-378 inhibitor. (b) Osteogenesis gene (Runx2, BMP2, Ocn, and Col1) was evaluated by qRT-PCR for angiogenesis enhancement at different time points after transfection including 2, 7, and 14 days. (c) ALP and alizarin red staining were enhanced and reduced by overexpression and inhibition of miR-378 expression, respectively. ^∗^*P* < 0.05 and ^∗∗^*P* < 0.01; *n* = 3.

To test the effect of miR378 on osteogenesis, we transfected BMMSCs with miR378 mimics. After 2-day, 7-day, and 14-day transfection of miR378 mimics, the miR378 level, assayed by qRTPCR, increased 90-fold at least compared with control (Figure 3(a)). Meanwhile, the osteogenetic gene expression including Runx2, BMP-2, ColI, and OCN was assayed by qRTPCR. The results showed that all osteogenetic gene expression increased significantly than control (Figure 3(b)), especially OCN expression was 60-fold more than control (Figure 3(b)). On the contrary, the miR378 level decreased 4–6-fold after the 2-day, 7-day, and 14-day transfections of miR378 inhibitor (Figure 3(a)). And osteogenetic gene expression got the same trend that all decreased to 2–5-fold, respectively, compared with control (Figure 3(b)).

After the 14-day transfection of miR-378 mimics in BMMSCs, ALP and alizarin red staining were significantly stronger than control. Contrarily, after the 14-day transfection of miR-378 inhibitor in BMMSCs, ALP and alizarin red staining were significantly weaker than control (Figure 3(c)).

### 3.3. Metrological Analysis

**Figure 4:**
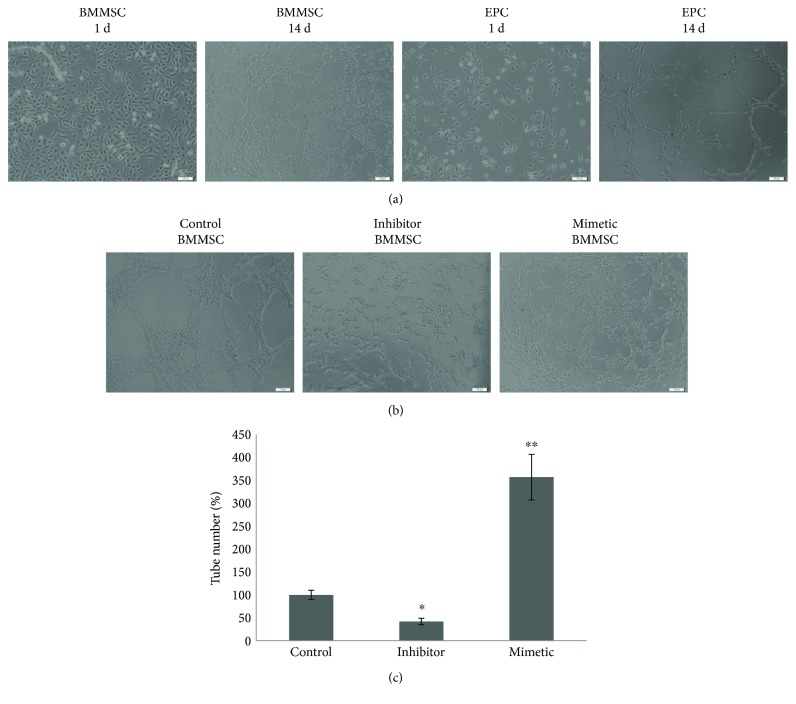
The effects of miR-378 on capillary tube formation of HUVECs on Matrigel. (a) Effects of capillary tube formation of HUVECs and EPC on Matrigel. (b) Effects of miR-378 on capillary tube formation of HUVECs on Matrigel. BMMSC that transfected with miR-378 mimetic, miR-378 inhibitor, and negative controls. After 14 days of incubation, the numbers of capillary tubes formed were counted (c). The experiment was repeated twice with comparable results. The untreated cells (control) were assigned to values of 100 and the results were presented as mean ± S.D. (*n* = 3). Significance: ^∗^*P* < 0.05 and ^∗∗^*P* < 0.01 versus control group.

Metrological analysis of BMMSC and EPC angiogenesis was performed using Matrigel. The BMMSC control group and the EPC control group showed no obvious vascular branch, node, and mesh. However, BMMSC group and EPC blood vessel group showed obvious vascular branch, node, and mesh, and for the BMMSC blood vessel group, the number of branches, nodes, and mesh was significantly higher than that of EPC (Figure 4(a)).

In order to detect the effect of miR-378 on the blood vessels, we transfected BMMSC cells with miR-378 mimetic, miR-378 inhibitor, or negative controls, respectively. After 14 days of transfection, the Matrigel was used to detect the number of branches, branches, nodes, mesh numbers, and mesh areas. The results showed that the number of branches and the length of branches were significantly higher than those of negative controls after transfection of miR-378 mimics. The number of nodes, mesh, and mesh area was also higher than that of negative controls. On the contrary, after transfection of miR-378 inhibitor, the number of branches, branches, nodes, mesh number, and mesh area was less than those of the negative controls (Figure 4(b)).

### 3.4. Increased Angiogenesis Related to miR378 Positively

**Figure 5:**
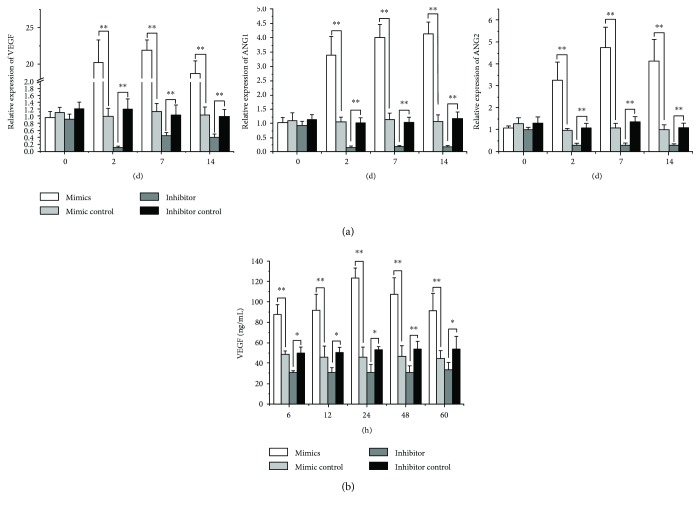
MiR-378 enhanced angiogenesis in vitro. BMMSCs were transfected with miR-378 mimics, miR-378 inhibitor, or the miR-negative control. (a) Angiogenesis gene (Ang1, Ang2, and VEGF) was evaluated by qRT-PCR for angiogenesis enhancement at different time points after transfection including 2, 7, and 14 days. (b) Overexpression of miR-378 significantly increased the VEGF protein secretion at 24 h, and levels increased through 60 h. ^∗^*P* < 0.05 and ^∗∗^*P* < 0.01; *n* = 3.

Besides osteogenetic gene, angioblastic gene VEGF and ANG-1 expression increased significantly after 2-day, 7-day, and 14-day transfection of miR-378 mimics, compared with control. And after the 2-day, 7-day, and 14-day transfection of miR-378 inhibitor, VEGF and ANG-1 expression decreased to 2-fold almost (Figure 5(a)).

After 6-hour, 12-hour, 24-hour, 48-hour, and 60-hour transfection of miR378 mimics or inhibitor in BMMSCs, VEGF secretion was assayed by ELISA. The results showed that VEGF secretion was significantly more than control after miR378 mimic transfection. Especially after 24-hour mimic transfection, the VEGF secretion reached the peak which was 6 times more than the control. And after the transfection of miR378 inhibitor in BMMSCs, VEGF secretion was significantly less than the control (Figure 5(b)).

**Figure 6:**
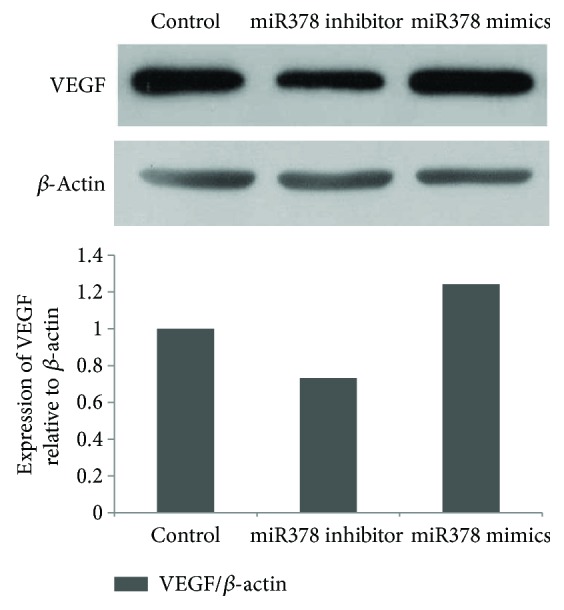
Determination of expression for detection of VEGF by Western blot immunoassay. VEGF was enhanced and reduced by overexpression and inhibition of miR-378 expression, respectively.

After BMMSCs were transfected with miR-378 mimics, miR-378 inhibitor, or negative controls, VEGF expression was analyzed by Western blot. The results showed that the expression level of miR-378 mimic group was significantly higher than that of negative control, and the expression of miR-378 inhibitor group was lower than that of negative control (Figure 6).

We promoted osteogenesis-angiogenesis coupling in BMMSCs through overexpression of miRNA378 in vitro and provided a potential gene tool choice for bone regeneration.

## 4. Discussion

miRNAs were demonstrated to participate in almost all the metabolic processes [14]. Therefore, miRNAs are researched as targeted gene therapy sites. We identified miR378 as angiogenesis-osteogenesis coupling in this article. In vitro experiment, angiogenesis and osteogenesis were positively regulated by miR378 in BMMSCs. miR378 is believed as an expected factor to promote bone through increasing angiogenesis and osteogenesis simultaneously.

Recently, increasing experiments were focused on the osteogenesis/angiogenesis and miRNA function. MiR214 was reported to suppress osteogenic and angiogenic differentiation by different downstream pathways involved in osterix and mitosis [17–20]. However, negative regulation would be seldom used for promotion in tissue regeneration. Some researchers found that miR566 could regulate angiogenesis by targeting the Von Hippel-Lindau tumor suppressor [21]. And miR-29b was proven to promote osteoblast differentiation by targeting several inhibitors of bone formation in vitro [22, 23]. MiR17 was believed to negatively regulate osteogenesis for bone tissue formation [24, 25]. Besides, miR26a was identified to regulate osteogenesis and angiogenesis for bone regeneration [26, 27]. In this work, we tried to identify miR378 as a choice of osteogenesis-angiogenesis coupling for bone regeneration.

miR378 has been reported as a key factor involved in sorts of cellular and organic metabolic processes. LEE found that miR378 could promote cell survival, tumor growth, and angiogenesis by targeting SuFu and Fus-1 expression [28]. It was reported miR-378 overexpression attenuated high glucose-suppressed osteogenic differentiation through targeting CASP3 and activating PI3K/Akt signaling pathway [29]. To verify miR378 as osteogenesis-angiogenesis coupling, we tested the osteogenetic gene markers including Runx2, OCN, ALP, and BMP2, in which osteogenetic gene played important roles in osteogenesis and bone formation [5, 6, 11, 30]. The osteogenetic markers showed significantly higher after miR378 transfection indicated BMMSCs to get stronger osteoblast capacity with overexpression of miR378. And ALP and alizarin red staining also demonstrated this conclusion.

Angiogenesis was highly associated with the function of angiogenic growth factors. VEGF was believed as the most important factor to promote proliferation and tube formation of vascular endothelial cells [8, 11, 12]. And angiotensin was also necessary to angiogenesis. The increased mRNA level of VEGF and ANG1/2 demonstrated that BMMSCs with overexpression of miRNA378 had enhanced angiogenic capacity.

We enhanced osteogenesis and angiogenesis simultaneously in BMMSCs through overexpression of miRNA378 in vitro and provided a potential gene tool choice for bone regeneration. And further delivery system and potential bone regeneration in vitro would be confirmed in the plan.

## Figures and Tables

**Table 1:** Primer sequences.

Gene	Sequences	
RUNX2	Forward	5′-CCCGTGGCCTTCAAGGT-3′
	Reverse	5′-CGTTACCCGCCATGACAGTA-3′

OCN	Forward	5′-CCCAGGCGCTACCTGTATCAA-3′
	Reverse	5′-GGTCAGCCAACTCGTCACAGTC-3′

COL I	Forward	5′-CCAGAAGAACTGGTACATCAGCAA-3′
	Reverse	5′-CGCCATACTCGAACTGGAATC-3′

ALP	Forward	5′-TAAGGACATCGCCTACCAGCTC-3′
	Reverse	5′-TCTTCCAGGTGTCAACGAGGT-3′

BSP	Forward	5′-GATTTCCAGTTCAGGGCAGTAG-3′
	Reverse	5′-CCCAGTGTTGTAGCAGAAAGTG-3′

VEGF	Forward	5′-TCGAGTCCGAGGGGGCCCAA-3′
	Reverse	5′-GAGCCAGGTCTCCCCGGCGTT-3′

ANG1	Forward	5′-GATGGTGATATTGCTGGGCT-3′
	Reverse	5′-GAGGAGCCTTTGTTGGAAG-3′

ANG2	Forward	5′-TGGAACCCATCTCCCGTTGA-3′
	Reverse	5′-AGACCAACAACAAAACGCCC-3′

*β*-Actin	Forward	5′-TGGCACCCAGCACAATGAA-3′
	Reverse	5′-CTAAGTCATAGTCCGCCTAGAGCA-3′
